# Invasive Nontyphoidal *Salmonella* Infection in a Patient with Early-Stage Chronic Lymphocytic Leukemia

**DOI:** 10.1155/2017/9091276

**Published:** 2017-10-29

**Authors:** Deepika Slawek, Yanina Dubrovskaya, Eddie Louie

**Affiliations:** Division of Infectious Diseases, NYU School of Medicine, 550 First Avenue NBV 16 South 5-13, New York, NY 10016, USA

## Abstract

We describe a case of a 72-year-old man with early-stage chronic lymphocytic leukemia (CLL) who presented with invasive nontyphoidal *Salmonella* (iNTS) infection, necrotizing pneumonia, and chronic infection of a hilar lymph node. Infection is a major cause of death in patients with CLL. Though few cases of iNTS infection associated with CLL have been described in the literature, to our knowledge this is the first reported case of iNTS-associated necrotizing pneumonia. Immunocompromised state in patients, even with early-stage CLL, likely predisposes them to invasive infection with intracellular organisms, such as *Salmonella* spp. In this case, successful treatment was achieved with prolonged course of intravenous followed by oral antibiotics without any surgical removal of infected focus.

## 1. Introduction

Nontyphoidal *Salmonella* (NTS) infections are common worldwide, usually causing a self-limited gastroenteritis lasting 5–7 days in immunocompetent hosts [1]. Invasive NTS (iNTS) infection, described as bacteremia and extraintestinal infection, is associated with young children and patients with malarial disease or HIV infection with CD4 count less than 500 [2] and has also been reported in immunocompromised patients other than those with HIV [3].

Here, we describe the presentation, treatment, and course of an unusual case of a patient with early-stage chronic lymphocytic leukemia (CLL) infected with *Salmonella enteritidis*, complicated by bacteremia, necrotizing pneumonia, and persistent infection of a hilar lymph node. To our knowledge, extraintestinal *Salmonella* infections have been described in the setting of CLL in only two other case reports [4, 5], neither of which involved focal pulmonary infection or treatment without surgical resection of the infected focus.

## 2. Case

A 72-year-old male with past medical history significant for CLL presented with four days of progressively worsening fever, cough productive of blood streaked sputum, and back pain. Almost two months prior to presentation, he had traveled to Southeast Asia with his family, during which he had a brief diarrheal illness that resolved within 24 hours without intervention. Four days prior to hospital admission, he noticed decreased exercise tolerance, followed by right-sided back pain, and progressively worsening cough and fever.

The patient's past medical history included a remote history of renal cell carcinoma with partial nephrectomy and CLL diagnosed two years prior to presentation with lymphocytosis and lymphadenopathy (Rai stage I)—baseline white blood cell count (WBC) of 12 K/uL. He had never required treatment for CLL prior to presentation and had immunoglobulin levels within normal limits several months prior to infection (IgA 119 mg/dL, IgG 730 mg/dL, IgM 51 mg/dL). He was otherwise well, not on any prescription medications, and very physically active.

On presentation to the hospital, he was febrile to 38.9°C and short of breath, with a WBC count of 30.2 K/uL (95% lymphocytes, 2% bands). Chest X-ray revealed an area of consolidation in the right upper lobe with associated area of cavitation. The patient was admitted and started on vancomycin, piperacillin-tazobactam, and azithromycin. Sputum culture collected at the time of admission returned positive for innumerable *Salmonella* species within 24 hours of collection; further, it was identified as *Salmonella enteritidis* when sent to the New York City Department of Health for serotyping. One set of blood cultures drawn on admission resulted positive in one out of two bottles four days after they were drawn, identified as *Salmonella enteritidis* and also serotyped by the New York City Department of Health. There was no growth from repeat blood cultures drawn one day following positive result while on empiric antibiotics. Susceptibilities performed by E-test showed *Salmonella enteritidis* susceptible to ceftriaxone, after which antibiotic regimen was changed to ceftriaxone 2 grams intravenous (IV) daily.

**Figure 1:**
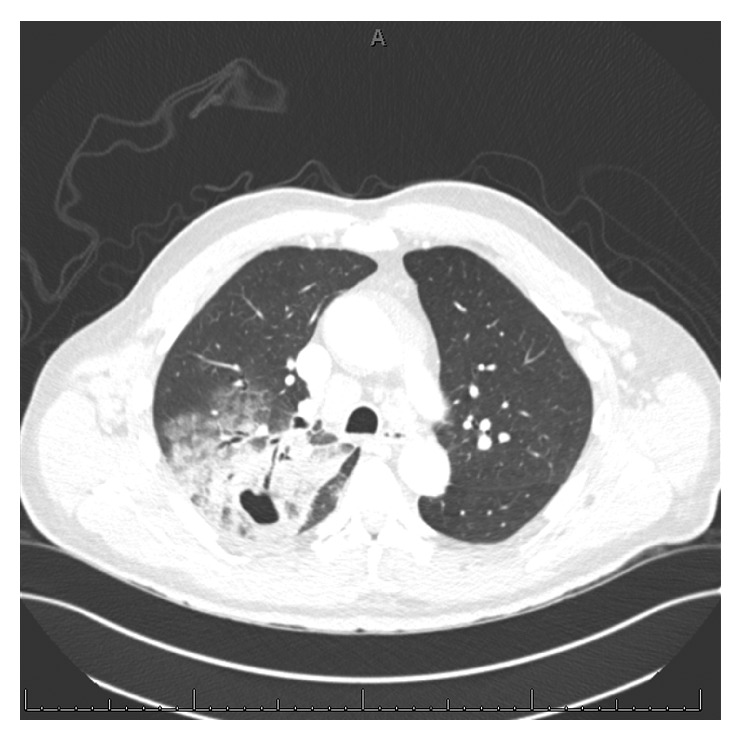
Contrast-enhanced computed tomography of the chest showing a cavitary mass with associated ground glass opacities and pneumonia.

Computed tomography of the chest performed in the first 24 hours of admission revealed multiple bilateral pulmonary emboli and a large right upper lobe cavitary mass with adjacent ground glass opacities and septal thickening, as well as moderate bilateral axillary, mediastinal, and upper abdominal adenopathy (Figure 1). Further microbiologic studies revealed positive BioFire FilmArray® Gastrointestinal Panel (BioFire Diagnostics, Salt Lake City, Utah, USA) for *Salmonella* sent five days after admission when the patient developed diarrhea, and stool culture sent at the same time with rare *Salmonella enteritidis*, also typed by the New York City Department of Health with the same susceptibilities.

**Figure 2:**
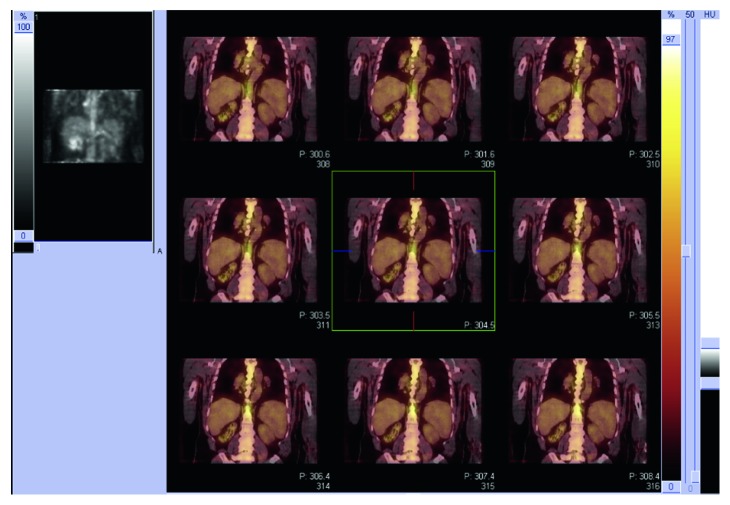
Gallium scan performed two months following completion of IV antibiotics showing intense radiotracer uptake in the right hilum corresponding with a known area of lymphadenopathy suggestive of an active process. No other areas of known lymphadenopathy show increased gallium uptake.

He defervesced within hours of administration of antibiotics and was treated with four weeks of ceftriaxone 2 grams IV daily. Follow-up imaging showed complete resolution of cavitary pneumonia. PET scan was performed for CLL monitoring one month after admission, showing cervical, axillary, hilar, mediastinal, upper abdominal, and iliac chain lymphadenopathy with low-grade metabolic activity, as well as uptake in the right upper lobe of the lungs indicating residual inflammation. During the following two weeks, the patient developed recurrent fevers for which he received GAMUNEX®-C (immune globulin injection [human], 10% caprylate/chromatography purified), but fevers persisted. Gallium scan was performed to evaluate for residual infection, which revealed strong radiotracer uptake in the right hilum with corresponding 6.3 × 4.2 cm lymphadenopathy seen on CT chest obtained at the same time (Figure 2), as well as mild uptake in the right upper lobe of the lung. He was started on cefpodoxime 200 milligrams orally twice daily, with resolution of fevers after one week of therapy, on which he continued for 4 months. Surgical excision for source control was not attempted due to difficult to access location of infected lymph node and patient's response to antibiotic therapy with no recurrent infection. He continues to be followed closely, with no evidence of recurrent or persistent salmonellosis on evaluation with clinical exam and laboratory testing.

## 3. Discussion

Infectious complications are known to be a major cause of morbidity and mortality in patients with CLL [6]. The pathogenesis of immunodeficiency and predisposition to infection in CLL are multifactorial, with primary CLL-related risk factors identified as hypogammaglobulinemia, defect in cell-mediated immunity, defective neutrophil function, increased length of disease (longer duration correlates with increased risk of severe infection), and advanced stage of disease [7]. Treatment-related risk factors include steroid-induced immune defects, induced T-cell defects from cell-mediated chemotherapy, and chemotherapy-related neutropenia [7].

Given the extent and severity of infection in the described patient, it is likely that there was predisposition to infection leading to this presentation. Our patient did not receive steroids or chemotherapy and had normal immunoglobulin levels, implying that the underlying defect was one in cell-mediated immunity. Cell-mediated defects in patients with CLL have been described as both primary defects and chemotherapy-induced defects [7]. Markers of T-cell exhaustion have been identified in patients with CLL, leading to a loss of T cell proliferation, impaired T cell cytotoxicity, and decreased cytokine production [8]. A variety of other functional defects of T cells in patients with CLL have been described including an increase in the number of T suppressor lymphocytes, reduction in number of T-helper cells, and secretion of soluble factors from CLL cells causing immune dysfunction [9–11]. Cell-mediated immunity is important in defense against *Salmonella* species, which are facultative intracellular organisms.

Patients with CLL presenting with iNTS infection have only been described in two other case reports. One case report was of a patient with early-stage CLL, who presented with *Salmonella* mycotic thoracoabdominal aortic aneurysm. The patient was treated with IV antibiotics and thoracic aneurysm repair with removal of involved aneurysm sac [4]. The case involved *Salmonella* superinfection of thyroid goiter following septicemia in a patient with undiagnosed CLL. The infection was treated with IV ceftriaxone as well as surgical excision of infected goiter. [5] In our case, successful treatment was achieved with prolonged antibiotics without any surgical removal of infected focus.

In conclusion, we have described a case of iNTS infection with necrotizing pneumonia and chronically infected hilar lymph node treated successfully with prolonged antibiotics. This case suggests that even early-stage CLL should be considered a risk factor for iNTS for patients with the appropriate exposure history.
